# Impact of Influenza on Children in a Referral Hospital in Mexico City: Clinical Burden and Predictors of Mechanical Ventilation

**DOI:** 10.3390/v17060771

**Published:** 2025-05-28

**Authors:** Rodolfo Norberto Jiménez-Juárez, Sarbelio Moreno-Espinosa, Alfonso Reyes-Lopez, Israel Parra-Ortega, Almudena Laris-Gonzalez, Daniela De la Rosa-Zamboni, Paola Guerra-de-Blas, Ana Estela Gamiño-Arroyo

**Affiliations:** 1 Department of Infectious Diseases, Federico Gómez Children’s Hospital of Mexico, Mexico City 06720, Mexico; almu_laris@hotmail.com; 2 Medica Sur Hospital, Mexico City 14059, Mexico; sarbelio.infecto@gmail.com; 3 Center for Economic and Social Studies in Health, Federico Gómez Children’s Hospital of Mexico, Mexico City 06720, Mexico; alfonso.reyes.lopez@outlook.com; 4 Clinical Laboratory Department, Federico Gómez Children’s Hospital of Mexico, Mexico City 06720, Mexico; i_parra29@hotmail.com; 5 Epidemiology Department, Dr. Eduardo Liceaga General Hospital of Mexico, Mexico City 06726, Mexico; rzdaniela@hotmail.com; 6 Research Unit on Infectious Diseases, Federico Gómez Children’s Hospital of Mexico, Mexico City 06720, Mexico; paolaguerrab@gmail.com; 7 The Mexican Emerging Infectious Disease Clinical Research Network, Mexico City 14050, Mexico; 8 Epidemiology Department, Federico Gómez Children’s Hospital of Mexico, Mexico City 06720, Mexico

**Keywords:** influenza, SARI, school-age children, Mexico, risk factors, mechanical ventilation

## Abstract

Influenza is a highly transmissible seasonal disease that significantly impacts public health worldwide, causing lower respiratory tract infections, numerous hospitalizations, and prolonged stays. However, data on its clinical burden in children in Latin America remain limited. This retrospective cohort study analyzed the demographic and clinical characteristics of children hospitalized with influenza in Latin America, stratified by age, and identified factors associated with mechanical ventilation. Medical records of children with severe acute respiratory infection associated with influenza were reviewed. Statistical analyses included chi square and Wilcoxon tests to compare groups, and Cox regression to identify predictors of mechanical ventilation. Among 212 cases, 46% of admissions were in school-age children; 93.4% had comorbidities. Influenza AH1N1 was more frequent in children <5 years and influenza H3N2 in children >5 years of age. The mechanical ventilation rate per age group was 24.1% among those <1 year, 29.8% in 1–4 years of age, 4.9% in 5–9 years of age, and 26.3% in children 10–18 years of age. Hypotension, paradoxical breathing, and nosocomial infection were identified as predictors for mechanical ventilation. These findings enhance the understanding of influenza’s clinical impact on pediatric populations, particularly in predicting severe outcomes requiring intensive care, and aid in developing strategies to mitigate its effects.

## 1. Introduction

Influenza is a highly contagious, vaccine-preventable respiratory illness capable of causing pandemics. During the 20th century, there were five influenza pandemics. The Spanish flu had the highest death toll, at between 20 and 50 million people [1]. The most recent flu pandemic occurred in 2009, known as the H1N1 pandemic, and caused a relatively low number of deaths, estimated at 413,500 [2].

There are around a billion cases of seasonal influenza annually, including 3–5 million cases of severe illness [3]. It is estimated that lower respiratory tract infections (LRTIs) due to influenza cause 9,459,000 hospitalizations and 81,536,000 days of hospitalization [4]. Influenza in children is responsible for 10% of hospitalizations due to LRTIs [5], and during the peaks of its activity, it causes an excess of hospitalizations (4–104/100,000) in children under 15 years of age, as well as 10–30% of excess in the use of antibiotics [6,7]. In Mexico, the flu season starts in week 40 and ends in week 20 of the following year, similar to other countries in the Northern Hemisphere, with activity peaks in December and January [8,9].

In Latin America, some studies report only rates of admission and death by age group; children under 1 year of age and persons over 65 are recognized as age groups with a risk of hospitalization and death. Nevertheless, the age groups are overly broad and the burden of influenza in children is not appreciated due to the lack of data in other pediatric age groups [10].

In the USA, the American Academy of Pediatrics (AAP) recommends universal immunization against influenza; however, in other lower middle-income and even upper middle-income countries, the recommendation is immunization only for high-risk children with complications or risk of death (<5 years old, pulmonary diseases, cancer, immunosuppression, HIV, solid organ transplant, cardiac diseases, obesity, or neurologic impairment) [11]. In Mexico, immunization against influenza includes children from 6 m to 5 years of age and older with comorbidities. Any effort to expand the age at which influenza vaccination is recommended requires local data on the clinical and economic burden of the disease in the groups of interest.

The primary objective of this study was to analyze the demographic and clinical characteristics of children admitted to the hospital for influenza stratified by age groups and their outcomes. The secondary objective was to identify predictors of mechanical ventilation (MV).

## 2. Materials and Methods

### 2.1. Study Population and Definitions

This retrospective cohort study included children hospitalized (at least 24 h in Emergency Room) to the Federico Gómez Children’s Hospital of Mexico (FGCHM) for influenza during five flu seasons from 2013 to 2018. The FGCHM is a tertiary referral hospital in Mexico City, serving pediatric patients from across the country. The hospital has a total of 220 beds, with 20 dedicated to the emergency room, 20 to the pediatric intensive care unit (PICU), and 30 to the neonatology unit.

To identify eligible patients, the molecular biology laboratory database was reviewed for cases of influenza detected via multiplex PCR. Respiratory virus detection by multiplex PCR is a standard care for children with severe acute respiratory infection (SARI) at this hospital, starting in 2013. All records of the subjects with detection of influenza by PCR and included only those with SARI, excluding those with nosocomial influenza (defined as symptom onset more than two days after admission) and for whom the reason for admission was another cause.

SARI was defined as an acute respiratory illness of recent onset with a manifestation of fever (≥38 °C), cough, and shortness of breath or difficulty breathing requiring hospitalization.

Data collected from patient records included age, sex, history of influenza vaccination (primary document was National Card of Vaccination; secondary document was admission evaluation), symptoms, blood count, viral coinfection, comorbidities, nosocomial infections different to nosocomial influenza (e.g., nosocomial sepsis), length of stay (LoS), admission to the PICU, MV, and death.

Nosocomial infections, also referred to as healthcare-associated infections (HAI), were defined as those infections acquired during the process of receiving health care that were not present during the time of admission [12]. In the current study, patients in this category were those admitted with influenza-related SARI (Influenza-SARI) who developed HAI during their hospital stay.

According to the World Health Organization, an influenza-like infection (ILI) is defined as an acute respiratory illness with a measured temperature ≥ 38 °C and cough with onset within the past 10 days [13,14]. Mexico’s definition is an individual with a history of fever or measured fever >38 °C, cough, and headache (irritability for <5 years) with one or more symptoms of rhinorrhea, coryza, arthralgias, myalgias, prostration, thoracic pain, abdominal pain, nasal congestion, and diarrhea [15].

MV was defined as requiring invasive respiratory support, which includes endotracheal intubation and ventilatory support via a mechanical ventilator [16]. Non-invasive respiratory support modalities, such as high-flow oxygen therapy, CPAP, or BiPAP, were not classified as MV in our analysis.

### 2.2. Microbiology

Samples were processed using an RT–PCR system with microarray visualization (CLART PneumoVir, Genomica, Madrid, Spain) capable of detecting adenovirus, bocavirus, coronavirus, rhinovirus, enterovirus, influenza virus A (subtypes AH3N2 and AH1N1), influenza virus B, metapneumovirus (subtypes A and B), influenza virus C, parainfluenza virus 1, 2, 3, and 4 (subtypes A and B), and respiratory syncytial virus type A (RSV-A) and B (RSV-B), with a sensitivity of 83.3–100% depending on the virus. In the case of influenza, the sensitivity and specificity were 91% and 99% for influenza A and 82% and 99% for influenza B, respectively [17]. Influenza B was classified into the Yamagata or Victoria lineages using PCR when RNA was available.

### 2.3. Ethics Statement

This study was conducted according to the guidelines of the Declaration of Helsinki and the use of the samples and data for this sub-study was approved by the Institutional Review Board (or Ethics Committee) of Hospital Infantil de México. This study is a product of protocols HIM/2017/132 and HIM/2018/015, registered and approved by the IRB committees of Federico Gómez Children’s Hospital of Mexico.

### 2.4. Statistical Analysis

Statistical analysis was performed with STATA 16.0. We estimated the frequency and percent of categorical variables. For quantitative variables, we estimated the mean with standard deviation or median with an IQR between the 25th and 75th percentiles and calculated rates of influenza admission per 100 discharges (n influenza/n discharges per month multiplied by 100). Our population was divided into age groups of <1 year, 1 to 4 years, 5 to 9 years, and 10 to 18 years. Analyses of associations of dichotomous variables were performed using the chi square or Fisher’s exact test. For continuous variables, the Kruskal–Wallis test was used. A survival analysis was performed to assess the time that MV was not needed in patients with influenza. Survival functions were estimated using the Kaplan–Meier actuarial method, stratified by age group. A Cox proportional hazards regression model was adjusted to assess the factors associated with the risk of MV in these patients. After adjusting a full model with different covariates, including demographic and clinical characteristics of patients, we decided to adjust a reduced model only with those covariates that were statistically significant and others, such as sex and age, as control variables. The proportional hazards assumption was tested based on the analysis of scaled Schoenfeld residuals.

Descriptive analysis included estimates of relative frequencies for categorial variables while for continuous variables we calculated central tendency and dispersion measures. Independence hypotheses between categorical variables were tested with the chi squared and Fisher exact tests. Additionally, the Kruskal–Wallis test was used to contrast differences between groups. The time-to-event analysis (also known as survival analysis) was used to estimate survival functions and hazard functions by means of the Kaplan–Meier method, the failure endpoint of interest being mechanical ventilation (MV). To evaluate the factors affecting the hazard of MV, a cox regression model was adjusted including demographic and clinical variables as regressors. The variable “nosocomial infection” was considered for inclusion in the Cox model only if present previously to mechanical ventilation. All procedures were performed with the Stata program version 16.0.

## 3. Results

**Figure 1:**
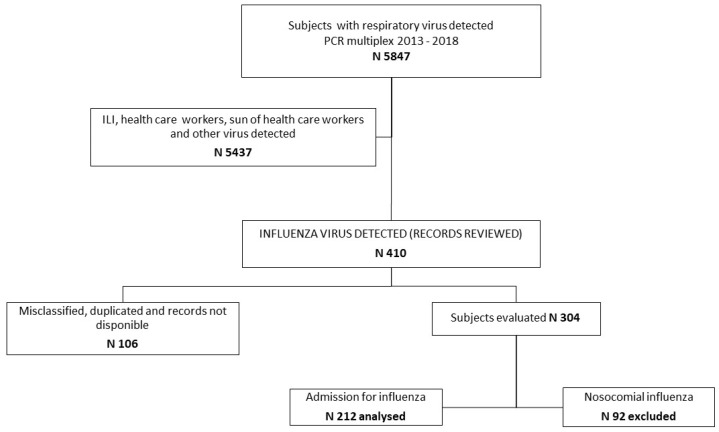
Study flow chart. Diagram for subject selection.

In five seasons of influenza (week 40 to week 20 of the following year), 5847 multiplex PCR tests to detect respiratory viruses were processed. In this database, we identified and reviewed 410 records of patients with influenza. After discarding duplicate and unavailable records and those corresponding to nosocomial influenza, 212 influenza cases were analyzed (Figure 1).

In the study group, 93.4% of children had comorbidities. Admissions for influenza were more frequent in the 1 to 4 years group (39.6%), followed by the 5–9 (28.8%) years and >10 years groups (17.9%); unexpectedly, the group of younger than 1-year-old group had the lowest frequency (13.7%) (Table 1). In total, 6.1% were vaccinated, 51.4% were unvaccinated, and 45.3% had no record of receiving the influenza vaccine on the National Vaccination Card (status unknown).

Children older than five years had higher frequencies of cancer (41.4% vs. 15.9%), immunodeficiencies (15.1% vs. 1.2%), and hematologic diseases (7.1% vs. 1.2%). Younger children (<5 years) showed greater prevalence of cardiovascular diseases (44.2% vs. 3%), pulmonary diseases (23.2% vs. 6.2%), and congenital malformations (30.4% vs. 9.5%). Rheumatologic disease was exclusively found in children older than 5 years (9.5% vs. 0%). Influenza AH1N1 was more frequent in children aged 1–4 years (41.7%) and influenza AH3N2 was more frequent in children aged ˃4 years (41.4%). Fifty-four children (25.5%) had viral coinfection, which was more frequent in children younger than 1 year (44.8%) (Table 1 and Table 2).

The most frequent symptoms were fever (79.3% in children under 1 year to 95.1% in the 5 to 9 years group) and cough. The distribution of signs and symptoms according to age group is presented in Table 3. ILI according to the WHO’s definition was present in 77.4% of children but in only 14.9% of children according to the Mexican definition.

**Figure 2:**
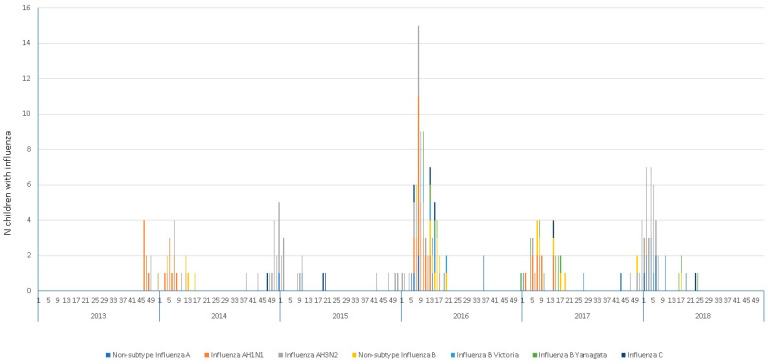
Seasonality of admissions for influenza 2013–2018 at the Federico Gómez Children’s Hospital of Mexico. Seasons 2016–2017 and 2017–2018 cocirculated influenza B lineages Yamagata and Victoria. Peaks of influenza activity found in January.

Influenza activity in our center is mostly observed from December to February, with a peak of activity in January (Figure 2). Usually, all subtypes of influenza tend to circulate during all seasons. In 2014, 2016, and 2017, influenza AH1N1 was more common, but in 2015 and 2018, influenza AH3N2 was more common. Out of the 48 children with influenza B, lineages were determined in 26 cases. Cocirculation of influenza B lineages Yamagata and Victoria was found in the 2016–2017 and 2017–2018 influenza seasons; in the 2013–2014 and 2014–2015 seasons, it was not possible to determine the lineage, and in 2015–2016, only Yamagata was identified. The highest rate of hospitalization for influenza was in the 2015–2016 season, with an average of 0.16/100 discharges in October–December 2015 and an increased rate of 4.70/100 discharges in February 2016.

**Figure 3:**
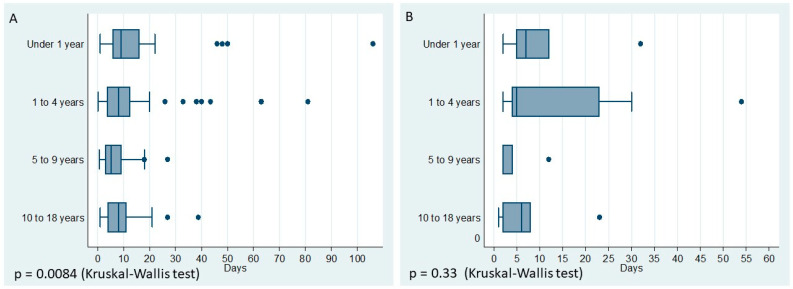
Length of stay per age group. (**A**) Length to stay for all hospitalizations; (**B**) length to stay for PICU.

The median LoS was 8 days (IQR 25th–75th 3.7–11.5). Children admitted to the PICU had a median LoS of 5 days (IQR 25th–75th 3–12). The results per age group are shown in Table 3 and Figure 3. In our study, 117 children received oseltamivir. However, in the bivariate analysis, we did not observe a significant effect of oseltamivir on the need for mechanical ventilation. Due to this lack of association, oseltamivir was not included in the multivariable model.

**Figure 4:**
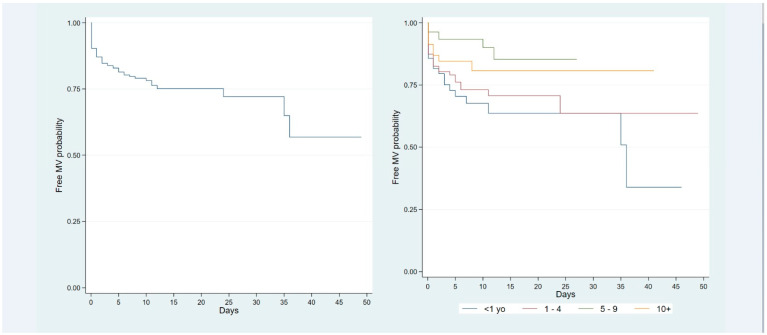
Kaplan–Meir curves for mechanical ventilation. (**A**) Free days of mechanical ventilation for all cohorts; (**B**) free days of mechanical ventilation per age groups.

MV was more prevalent among infants younger than 1 year old and less prevalent in those aged 5–9 years old (Table 3). Survival analyses show the initial rapid decline in survival function caused by patients requiring urgent MV (Figure 4). On the first day of admission, the probability of not needing MV was 88.4%. From the second day, the survival function continued to decrease but slowly plateaued at 77.8% (Figure 4). The overall survival curve shows a general trend of decreasing probability of remaining free from MV over time. This indicates that the likelihood of requiring MV increases as patients stay in the hospital longer.

The survival functions by seasonal flu show important differences between the 2012–2013 season (reference category) and 2014–2015, 2015–2016, and 2017–2018, in which children have a lower risk of needing MV (Table 4). Hypotension and paradoxical breathing resulted in a more than threefold increase in the risk of MV, whereas nosocomial infection resulted in an almost twofold increase in the risk of MV; there were 25 nosocomial infections, but the 10 prior to MV were not included in the Cox model. Of the 15 included there were 13 sepsis cases and two pneumonia cases; all these factors were statistically significant. In contrast, having an immunodeficiency did not increase the risk of MV in patients with influenza, most likely because this condition receives close surveillance by pediatricians (Table 4). The age group did not impact in the risk of MV. The global test of the proportional hazards assumption resulted in a *p* = 0.19, which does not violate this assumption.

Mortality was found in only six cases, five associated with influenza AH1N1 and one with non-subtype influenza A. Five deaths occurred on days 2–8, one death on day 12, and one death on day 22 after admission; four were attributable to nosocomial infection (two nosocomial sepsis cases and two nosocomial pneumonia cases). Three children were less than 1 year old, one child was 1.4 years old, one child 2.3 years old, and one child was 5.6 years old (Table 3). All children had comorbidities, three with a neuromuscular disorder (cerebral palsy and neuromuscular dystrophy), one with hematology neoplasm, one with pulmonary disorder, and one with congenital heart disease.

## 4. Discussion

Influenza is a highly transmissible seasonal disease that imposes a substantial burden on public health worldwide [18]. While the impact of influenza on children has been extensively studied in high-income countries, there is a significant lack of information regarding its clinical characteristics and burden in middle-income countries, particularly in Latin America. This study represents one of the largest investigations of severe acute respiratory infection (SARI) associated with influenza in children in Latin America, providing valuable insights into the disease burden and clinical presentation of SARIs in this specific region. Additionally, this study aimed to identify factors that predict the need for MV, a critical measure of disease severity.

One of the challenges in influenza surveillance among children is the sensitivity of operational definitions, particularly for SARIs. Other studies, one in India and one in Mexico, have also showed that a significant number of cases of infection with influenza are missed by strictly following the WHO case definitions [19,20]. This discrepancy is significant for the surveillance and medical care of SARIs for influenza, as physicians may not have an immediate etiology, potentially delaying treatment for influenza.

Our observations revealed that most children who tested positive for influenza had underlying diseases and a low percentage of influenza immunization. This finding distinguishes our study from previous research, where most hospitalized children with influenza were reported to have no comorbidities [6,21]. Exceptions include studies by Launes, who reported a comorbidity rate of 30% in Spain [22], and Böncüoğlu, who reported 39% [23]. This discrepancy can be attributed to the fact that our study was conducted in a center providing care to high-risk populations.

Vaccine coverage for influenza vaccination in Mexico has varied in the application of the complete vaccination schedule in children, from over 96% in 2009–2018 to 76% in 2019, 33% in 2020, and 86.6% in 2022, as reported by the Pan-American Health Organization (PAHO) and the National Health and Nutrition Survey (ENSANUT, Spanish acronym) [24,25]; however, proof of influenza vaccine coverage in our population was extremely low, necessitating a nominal census to accurately estimate immunization coverage. In the United States, vaccination coverage among children under 5 years of age who visited the emergency room due to influenza was only 6% during the period of 2000–2004, but increased to 38% by 2010–2011 [26]. In Spain, reports indicated that among hospitalized children, immunization coverage stood at 58%, while for children with chronic diseases, it was 27% [27]. Low vaccine coverage could be explained by recall bias for vaccination status, which we attempted to reduce by using the National Card of Vaccination as the primary information document and the admission evaluation as a secondary option. Despite these efforts, 45.3% of participants did not know their vaccination status.

Another possible explanation is the effectiveness of the influenza vaccine against hospitalization. Recent findings from a study in the USA reported a global effectiveness of 75% in preventing life-threatening cases of influenza, although this effectiveness was lower (47% (95% IC −21 to 77%)) when influenza AH1N1 was mismatched [28]. Efforts should be directed towards improving vaccination coverage in children with underlying diseases, in addition to conducting vaccine effectiveness studies in children with comorbidities. Governments should consider expanding recommendations for influenza immunization, similar to the universal approach in the USA. An economic evaluation in a subgroup of our patients reported a median cost of $9710 USD (IQR $2442–$9710 USD), suggesting that it is cost-effective to expand vaccination recommendations. Additionally, Falcon-Lezama et al. conducted a cost-effectiveness study to expand vaccination recommendations to schoolchildren in Mexico, finding that this strategy is cost-saving, estimated to save 111.9 million USD [29]. Recently our group examined the economic burden of influenza-related severe acute respiratory infections (SARIs) using this dataset. Our findings highlighted the substantial financial strain influenza places on the healthcare system. Children with underlying health conditions incurred higher costs, and those over 10 years of age consumed significant resources despite being often overlooked in immunization programs [30].

It has been reported that Hispanic or Latino populations have been identified as having higher rates of hospitalization from influenza [31]. Notably, in this study, children between 5–9 years of age were the age group with the second-highest number of hospitalizations, highlighting the significant burden of influenza in this age group. Unlike other studies, in our study, the age group that had the lowest percentage of hospitalizations due to influenza was the <1 year group. Traditionally, this group has the highest frequency of hospitalizations in addition to other unfavorable outcomes [21,32,33,34].

The seasonality pattern we observed aligns with the findings reported at the national level by the WHO’s FluNet. We noted increased influenza activity towards the end of December and the beginning of January, extending until March [35].

We found cocirculation of two lineages of influenza B during two out of four seasons. Circulating influenza subtypes are similar to those reported by WHONET and the Directorate General of Epidemiology (DGE), except for the 2015–2016 season when influenza A H1N1 and AH3N2 cocirculated in our hospitalized cases in almost equal proportions. Furthermore, we identified cocirculation of the Victoria and Yamagata lineages in the last two influenza seasons evaluated. These data support the importance of including the tetravalent influenza vaccine in the universal immunization scheme.

Additionally, influenza C was detected with our platform in 4.2% of children with SARI. Although this type of influenza virus is not frequently reported, it requires confirmation through sequencing [36,37]. One study in Korea reported 1.3% of children with influenza C, with clinical expression as an upper respiratory infection and no cases of pneumonia [37]. Additionally, there are several reports which have detected influenza C among children with ILIs and patients hospitalized for severe acute respiratory infection [36,37,38].

During the COVID-19 pandemic, there was a decrease in the epidemiological curve before the end of the 2019–2020 influenza season, followed by a resurgence during the 2021–2022 season [39]. Subsequently, there were more interseason cases than expected. Notably, during the 2022–2023 season, we observed a higher number of cases compared to the pre-pandemic era. The future behavior of influenza in the coming years remains uncertain, both in terms of its magnitude during typical seasons and the potential occurrence of interseason cases. The dataset showed here, collected over 9 years, provides valuable information about the prevalence of influenza before the 2019 COVID-19 pandemic, which can help determine COVID-19-specific effects. This may help future decision-making about public health policies regarding disease prevention (including vaccination), the administration of health resources, and areas for focus in future research.

An important consideration is the challenge of clinically differentiating influenza from SARS-CoV-2 in children when they are cocirculating. Diagnostic tests are necessary to determine the appropriate placement of children within the hospital and to guide the use of specific antiviral treatments. This distinction is particularly crucial for populations at risk of severe disease associated with either of these two viruses [40].

In this study, we found twenty-one percent of children with influenza needed MV. Cummings performed a study in US and they found the need of MV in 5.1% of children with community-acquired pneumonia caused by influenza and in 19.8% in those with nosocomial infection [41]. MV has been evaluated in a few studies; in Brazil, comorbidities and viral coinfection were risk factors for MV [42]. In the USA, a multicenter study reported that 39% of children had comorbidities, and the risk factors for MV were comorbidities and bacterial coinfection [43], similar to Australia, where children with comorbidities have an increase odd of MV, ICU admission, and death [44].

It is important to be able to identify children who will require MV. In our cohort, we found paradoxical breathing, nosocomial infection, and hypotension are predictors for MV in children with an underlying disease. Ghimire and colleagues evaluated risk factors for respiratory failure in a registry of children hospitalized for influenza: predictors were congenital heart diseases, asthma, congenital respiratory anomalies, congenital musculoskeletal anomalies, and chromosomal anomalies [45]. Kamidani et al. identified that patients with abnormal upper airway, neurologic, or neuromuscular diseases had increased odds of requiring MV [33]. In our study, children less than 1 year of age did not show an increased risk of MV, whereas children over 10 years of age had a higher risk for MV. This could potentially be attributed to the presence of underlying diseases among the children, which might have influenced the outcomes, although other studies have also found an increased risk for MV in children >12 and >13 years [33,43].

Moreover, our study examined viral coinfection as a potential risk factor for MV. Surprisingly, viral coinfection was not found to be a significant risk factor, in contrast with the findings of a study performed in Brazil using direct immunofluorescence assay for evaluate viral coinfection [42]. The direct immunofluorescence assay had limited sensitivity for detecting other respiratory viruses and may have only identified children with higher viral loads, unlike the more sensitive RT-PCR. Further studies are needed to corroborate this finding.

Regarding the potential impact of antiviral use in the risk of MV, in our study, although 117 children received oseltamivir, bivariate analysis revealed no significant effect of the antiviral on the need for mechanical ventilation. Due to this lack of association, oseltamivir was not included in the multivariable model. This finding aligns with a systematic review and meta-analysis by Gao et al. in 2024 which also indicated that while oseltamivir and peramivir might reduce the duration of hospitalization for patients with severe seasonal influenza, the evidence regarding their impact on mortality and progression to invasive mechanical ventilation remains unclear [46]. These results suggest that while oseltamivir may offer certain benefits in managing influenza in children, its role in preventing severe respiratory complications requiring mechanical ventilation remains unclear.

Deaths occurred across all age groups, but our data do not allow us to evaluate risk factors for death. Tuckerman’s meta-analysis on children with chronic diseases found that this group had the greatest risk for hospitalization due to influenza; however, it did not find an increased risk of MV or death compared to children without chronic diseases [47].

Potential biases introduced by the retrospective design of this study include incomplete vaccination records, with 45.3% of cases lacking status data. Additionally, the large percentage of children with comorbidities included in one single center may limit the generalizability of the findings to both children without comorbidities and healthcare settings beyond this referral center. While our hospital serves as a referral center, variations in healthcare access and clinical practices exist across different regions. Our study did not evaluate bacterial coinfection, which is a limitation and potential bias that could impact our multivariate analyses, because many cases of influenza-like illness (ILI), particularly those requiring hospitalization, may involve bacterial secondary infections, which could impact disease severity and the need for MV. Future studies should incorporate systematic bacterial pathogen assessments, such as blood cultures and respiratory cultures, to better differentiate primary viral infections from secondary bacterial infections and their impact on patient outcomes.

Some of the strengths of this study include the number of hospitalized children in the sample. This sample size is comparable to recent studies in the field. This robust sample size enhances the statistical power and strengthens the reliability of the findings. Another significant strength is this study’s ability to evaluate the risk factors associated with MV. Another key strength of our study is the use of laboratory-confirmed influenza diagnoses, rather than relying solely on ICD-10 codes from administrative databases. This approach enhances the accuracy of our findings and allows for a more precise analysis of influenza strains circulating in the population.

## 5. Conclusions

In conclusion, there continues to be a substantial burden of influenza-related hospitalizations in all children after the 2009 H1N1 pandemic. This study provides insights on the clinical impact of influenza that are crucial for managing admissions to the pediatric intensive care unit (PICU), the need for MV, and the length of stay (LoS). Our findings indicate that children aged 10–18 years with severe acute respiratory infections (SARIs) have a higher likelihood of requiring MV. Community hospitals lacking MV capabilities should consider early referrals of children exhibiting hypotension or paradoxical breathing after initial stabilization.

These insights provide valuable evidence to inform clinical practice, guide risk assessment, and support the development of targeted interventions aimed at improving patient outcomes.

Efforts to increase flu vaccination rates among children must be intensified. Additionally, it is essential to investigate the reasons behind the low immunization rates among children who are eligible for vaccination.

## Figures and Tables

**Table 1:** Base characteristics of children by age group.

Variable	All Patients (n= 212)	Under 1 Year (n = 29)	1 to 4 Years (n = 84)	5 to 9 Years (n = 61)	10 to 18 Years (n = 38)	*p* Value
Female, n (%)	99 (46.7)	14 (48.3)	33 (39.3)	30 (49.2)	22 (57.9)	0.269
Comorbidities, n (%)	198 (93.4)	26 (89.7)	77 (91.7)	58 (95.1)	37 (97.4)	0.517
Pulmonary disease, n (%)	30 (15.2)	7 (26.9)	17 (22.1)	4 (6.9)	2 (5.4)	0.014
Allergic disease, n (%)	17 (8.6)	0 (0.0)	7 (9.1)	9 (15.5)	1 (2.7)	0.057
Endocrinologic disease, n (%)	17 (8.6)	3 (11.5)	6 (7.8)	3 (5.2)	5 (13.5)	0.461
Immunodeficiencies, n (%)	66 (33.3)	1 (3.8)	18 (23.4)	26 (44.8)	21 (56.8)	<0.001
Renal disease, n (%)	18 (9.1)	1 (3.8)	8 (10.4)	4 (6.9)	5 (13.5)	0.513
Rheumatologic disease, n (%)	8 (4.0)	0 (0.0)	0 (0.0)	2 (3.)	6 (16.2)	<0.001
Gastrointestinal disease, n (%)	20 (10.1)	5 (19.2)	7 (9.1)	5 (8.6)	3 (8.1)	0.526
Hematologic disease, n (%)	7 (3.5)	1 (3.8)	0 (0.0)	2 (3.4)	4 (10.8)	0.014
Congenital malformations	40 (20.2)	10 (38.5)	21 (27.3)	8 (13.8)	1 (2.7)	0.001
Cancer, n (%)	59 (27.8)	0 (0.00)	18 (21.4)	24 (39.3)	17 (44.7)	< 0.001
Cardiovascular disease, n (%)	37 (17.5)	14 (48.3)	20 (23.8)	3 (4.9)	0 (0.0)	< 0.001
Vaccine vs. influenza, n (%)	13 (6.1)	1 (3.5)	7 (8.3)	3 (4.9)	2 (5.3)	0.163
Influenza vaccine not registered, n (%)	96 (45.3)	10 (34.5)	31 (36.9)	35 (57.4)	20 (52.6)	0.163
Nonsubtype influenza A, n (%)	21 (9.9)	4 (13.8)	8 (9.5)	4 (6.6)	5 (13.2)	0.564
Influenza AH1N1, n (%)	61 (28.8)	12 (41.4)	35 (41.7)	10 (16.4)	4 (10.5)	< 0.001
Influenza AH3N2, n (%)	72 (33.9)	2 (6.9)	26 (30.9)	28 (45.9)	16 (42.1)	0.001
Influenza B, n (%)	50 (23.6)	7 (24.1)	14 (16.7)	16 (16.2)	13 (34.2)	0.311
Influenza C, n (%)	9 (4.2)	2 (6.9)	1 (1.2)	4 (6.6)	2 (5.3)	0.199
Viral Coinfection, n (%)	54 (25.5)	13 (44.8)	18 (21.4)	12 (19.7)	11 (28.9)	0.061

**Table 2:** Coinfection with other respiratory viruses.

Coinfection Viruses	Under 1 Year (n = 13)	1 to 4 Years (n = 18)	5 to 9 Years (n = 12)	10–18 Years (n = 11)
*Influenza A—Influenza AH1N1*	0 (0.00)	1 (1.19)	0 (0.00)	0 (0.00)
*Influenza A—Influenza B Victoria*	0 (0.00)	0 (0.00)	1 (1.64)	0 (0.00)
*Influenza A—RSVB*	3 (10.34)	2 (2.38)	0 (0.00)	2 (5.26)
*Influenza A—RSVA*	0 (0.00)	0 (0.00)	1 (1.64)	0 (0.00)
*Influenza A—Rhinovirus*	1 (3.45)	1 (1.19)	0 (0.00)	1 (2.63)
*Influenza A—Metapneumovirus B*	1 (3.45)	1 (1.19)	0 (0.00)	0 (0.00)
*Influenza AH1N1—RSVA*	3 (10.34)	2 (2.38)	0 (0.00)	0 (0.00)
*Influenza AH1N1—Parainfluenza 3*	0 (0.00)	1 (1.19)	0 (0.00)	0 (0.00)
*Influenza AH1N1—Metapneumovirus B*	0 (0.00)	1 (1.19)	0 (0.00)	0 (0.00)
*Influenza AH3N2—RSVA*	0 (0.00)	1 (1.19)	1 (1.64)	0 (0.00)
*Influenza AH3N2—RSVB*	0 (0.00)	3 (3.57)	2 (3.28)	1 (2.83)
*Influenza AH3N2—Parainfluenza 4*	0 (0.00)	0 (0.00)	0 (0.00)	1 (2.63)
*Influenza AH3N2—Rhinovirus*	0 (0.00)	1 (1.19)	0 (0.00)	1 (2.63)
*Influenza AH3N2—Bocavirus*	0 (0.00)	1 (1.19)	1 (1.64)	1 (2.63)
*Influenza AH3N2—Adenovirus*	0 (0.00)	0 (0.00)	0 (0.00)	1 (2.63)
*Influenza B—RSVA*	1 (3.45)	1 (1.19)	1 (1.64)	0 (0.00)
*Influenza B—RSVB*	1 (3.45)	1 (1.19)	1 (1.64)	0 (0.00)
*Influenza B—Parainfluenza 3*	0 (0.00)	1 (1.19)	0 (0.00)	0 (0.00)
*Influenza B—Rhinovirus*	1 (3.45)	0 (0.00)	0 (0.00)	0 (0.00)
*Influenza B—Metapneumovirus B*	0 (0.00)	0 (0.00)	1 (1.64)	0 (0.00)
*Influenza B—Bocavirus*	1 (3.45)	0 (0.00)	0 (0.00)	1 (2.63)
*Influenza B—Coronavirus 229E*	0 (0.00)	0 (0.00)	0 (0.00)	1 (2.63)
*Influenza B Yamagata—RSVB*	0 (0.00)	1 (1.19)	0 (0.00)	0 (0.00)
*Influenza Yamagata—Rhinovirus*	1 (3.45)	0 (0.00)	0 (0.00)	0 (0.00)
*Influenza B Victoria—Parainfluenza 1*	1 (3.45)	0 (0.00)	0 (0.00)	0 (0.00)
*Influenza B Victoria—Metapneumovirus B*	0 (0.00)	0 (0.00)	1 (1.64)	0 (0.00)
*Influenza B Victoria—Coronavirus NL63*	1 (3.45)	0 (0.00)	0 (0.00)	0 (0.00)
*Influenza B Victoria—Adenovirus*	1 (3.45)	0 (0.00)	0 (0.00)	0 (0.00)
*Influenza C—RSVB*	0 (0.00)	1 (1.19)	1 (1.64)	0 (0.00)
*Influenza C—Parainfluenza 3*	0 (0.00)	0 (0.00)	1 (1.64)	0 (0.00)
*Influenza C—Parainfluenza 4*	1 (3.45)	0 (0.00)	0 (0.00)	0 (0.00)
*Influenza AH1N1—RSVA—RSVB*	2 (6.90)	0 (0.00)	0 (0.00)	0 (0.00)
*Influenza AH1N1—RSVA—Parainfluenza 3*	0 (0.00)	1 (1.19)	0 (0.00)	0 (0.00)
*Influenza AH1N1 -RSVA—Metapneumovirus B*	0 (0.00)	1 (1.19)	0 (0.00)	0 (0.00)
*Influenza AH3N2—RSVA—RSVB*	0 (0.00)	0 (0.00)	1 (1.64)	0 (0.00)
*Influenza AH3N2 -RSVB—Rhinovirus*	0 (0.00)	1 (1.19)	0 (0.00)	0 (0.00)
*Influenza AH3N2 -Rhinovirus—Bocavirus*	0 (0.00)	0 (0.00)	0 (0.00)	1 (2.63)
*Influenza B—RSVA—RSVB*	1 (3.45)	1 (1.19)	0 (0.00)	0 (0.00)
*Influenza B—RSVA—Metapneumovirus B*	0 (0.00)	0 (0.00)	1 (1.64)	0 (0.00)
*Influenza B—Rhinovirus—Bocavirus*	1 (3.45)	0 (0.00)	0 (0.00)	0 (0.00)
*Influenza B Victoria—Coronavirus NL63—Adenovirus*	1 (3.45)	0 (0.00)	0 (0.00)	0 (0.00)

**Table 3:** Symptoms, signs, and outcomes in children by age group.

Variable	Under 1 Year (n = 29)	1 to 4 Years (n = 84)	5 to 9 Years (n = 61)	10 to 18 Years (n = 38)	*p* Value
Fever, n (%)	23 (79.3)	72 (85.7)	58 (95.1)	34 (89.5)	0.119
Cough, n (%)	23 (79.3)	69 (82.1)	55 (90.2)	31 (81.6)	0.412
Irritability, n (%)	11 (37.9)	14 (16.7)	2 (3.3)	2 (5.3)	<0.001
General discomfort, n (%)	6 (20.7)	12 (14.3)	14 (22.9)	5 (13.2)	0.468
Respiratory distress, n (%)	20 (68.9)	55 (65.5)	18 (29.5)	6 (15.8)	<0.001
Myalgia, n (%)	NA	2 (2.4)	6 (9.8)	2 (5.3)	0.131
Odynophagia, n (%)	1 (3.5)	4 (4.8)	8 (13.1)	5 (13.5)	0.156
Fatigue, n (%)	3 (10.3)	11 (13.1)	8 (13.1)	4 (10.5)	0.981
Conjunctival redness, n (%)	0 (zero)	6 (7.1)	4 (6.6)	3 (7.9)	0.542
Sneeze, n (%)	0 (zero)	6 (7.1)	3 (4.9)	3 (7.9)	0.509
Headache, n (%)	2 (6.9)	3 (3.6)	11 (18.0)	11 (28.9)	0.001
Nausea or vomiting, n (%)	9 (31.0)	15 (17.9)	11 (18.0)	4 (10.5)	0.212
Diarrhea, n (%)	5 (17.2)	13 (15.5)	3 (4.9)	1 (2.6)	0.036
**Laboratory tests**					
Leukocytes, mean (SD)	12,600 (7682)	9000 (6392)	6149 (4618)	7039 (8871)	0.3128
Lymphocyte percentage, mean (SD)	36.11 (16.76)	31.49 (18.69)	28.27 (18.05)	28.44 (23.96)	0.3128
Neutrophil percentage, mean (SD)	49.06 (18.80)	51.71 (20.46)	54.38 (25.03)	59.62 (22.97)	0.9860
Platelets, mean (SD)	288,481 (144,607)	244,228 (159,478)	224,377 (134,048)	186,921 (125,779)	0.9860
**Outcomes**					
Admitted to PICU, n (%)	5 (17.2)	15 (17.9)	6 (9.8)	10 (26.3)	0.297
Mechanical ventilation, n (%)	7 (24.1)	25 (29.8)	3 (4.9)	10 (26.3)	<0.001
Deaths, n (%)	3 (10.3)	2 (2.4)	1 (1.6)	0 (zero)	0.135
Length of stay in a hospital, days (IQR 25th–75th)	25 (6–16)	8 (3.66–12.81)	5.22 (3–9)	8 (4.5–12.5).	
Length of stay in a PICU, days (IQR 25th–75th)	8 (5–12)	5 (4–23)	2 (2–4)	6 (3–8)	

**Table 4:** Predictors of mechanical ventilation.

Predictors	Hazard Ratio	95% Confidence Interval	*p* Value
Sex	2.22	0.98–5.06	0.057
Age groups (ref: 5–9)			
<1 years old	0.89	0.19–4.18	0.89
1–4	2.16	0.60–7.84	0.24
≥10 years	4.38	0.85–22.67	0.78
Influenza season (ref: 2012–2013)			
2013–2014	0.12	0.01–1.22	0.073
2014–2015	0.05	0.002–0.67	0.024
2015–2016	0.09	0.008–0.99	0.049
2016–2017	0.12	0.009–1.36	0.086
2017–2018	0.02	0.0006–0.33	0.008
Hypotension	3.27	1.30–8.24	0.012
Paradoxical breathing	6.79	2.49–18.46	<0.001
Nosocomial Infection	2.78	1.10–7.01	0.03
Immunodeficiency	0.34	0.08–1.42	0.14

## Data Availability

The raw data supporting the conclusions of this article will be made available by the authors on request.
